# 1-(4-Methyl­phenyl­sulfon­yl)-5-phenyl-4,5-dihydro-1*H*-pyrazole

**DOI:** 10.1107/S1600536811025001

**Published:** 2011-07-02

**Authors:** Jie Li, Wei Zhao

**Affiliations:** a Medical College, Zhejiang University City College, Hangzhou 310015, Zhejiang, People’s Republic of China

## Abstract

The title compound, C_16_H_16_N_2_O_2_S, was synthesized by the reaction of 5-phenyl-4,5-dihydro-1*H*-pyrazole and 4-methyl­benzene-1-sulfonyl chloride. The five-membered pyrazoline ring is nearly planar, with a miximum deviation of 0.078 (2) Å.

## Related literature

For the pharmacological properties of pyrazoline derivatives, see: Goodell *et al.* (2006 ▶); Park *et al.* (2005 ▶); Shaharyar *et al.* (2006 ▶); Suresh *et al.* (2009 ▶).
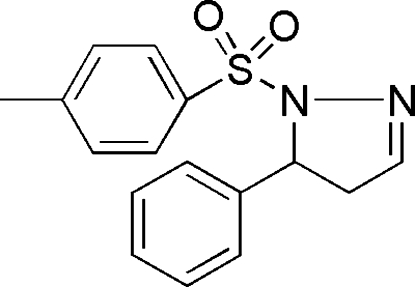


## Experimental

### 

#### Crystal data

C_16_H_16_N_2_O_2_S
*M* *_r_* = 300.37
Orthorhombic, 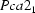

*a* = 19.2938 (7) Å
*b* = 6.0438 (2) Å
*c* = 12.9812 (5) Å
*V* = 1513.71 (10) Å^3^
*Z* = 4
Mo *K*α radiation
μ = 0.22 mm^−1^
*T* = 293 K
0.32 × 0.28 × 0.25 mm

#### Data collection

Oxford Diffraction Xcalibur Atlas Gemini ultra diffractometer
Absorption correction: multi-scan (*CrysAlis PRO*; Oxford Diffraction, 2010 ▶) *T*_min_ = 0.933, *T*_max_ = 0.947
5286 measured reflections
2168 independent reflections
1870 reflections with *I* > 2σ(*I*)
*R*_int_ = 0.028

#### Refinement

*R*[*F* ^2^ > 2σ(*F* ^2^)] = 0.034
*wR*(*F* ^2^) = 0.081
*S* = 1.05
2168 reflections
191 parameters
1 restraint
H-atom parameters constrained
Δρ_max_ = 0.15 e Å^−3^
Δρ_min_ = −0.26 e Å^−3^
Absolute structure: Flack (1983 ▶), 711 Friedel pairs
Flack parameter: 0.00 (8)

### 

Data collection: *CrysAlis PRO* (Oxford Diffraction, 2010 ▶); cell refinement: *CrysAlis PRO*; data reduction: *CrysAlis PRO*; program(s) used to solve structure: *SHELXS97* (Sheldrick, 2008 ▶); program(s) used to refine structure: *SHELXL97* (Sheldrick, 2008 ▶); molecular graphics: *OLEX2* (Dolomanov *et al.*, 2009 ▶); software used to prepare material for publication: *OLEX2*.

## Supplementary Material

Crystal structure: contains datablock(s) global, I. DOI: 10.1107/S1600536811025001/bv2186sup1.cif

Structure factors: contains datablock(s) I. DOI: 10.1107/S1600536811025001/bv2186Isup2.hkl

Supplementary material file. DOI: 10.1107/S1600536811025001/bv2186Isup3.cml

Additional supplementary materials:  crystallographic information; 3D view; checkCIF report

## Figures and Tables

**Fig. 1.**
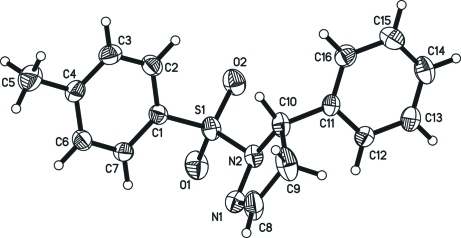
The molecular structure of title compound

## supplementary crystallographic information

### Comment

5-Phenyl-1-tosyl-4,5-dihydro-1*H*-pyrazoles are a key intermediates which can be used to synthesize pyrazoline derivatives, which are well known for their versatile pharmacological activities such as antitumor (Park *et al.*, 2005), antibacterial (Shaharyar *et al.*, 2006), antifungal (Goodell *et al.*, 2006), antiviral, antiparasitic, anti-tubercular and insecticidal agents (Suresh *et al.*, 2009). The title compound is one of these compounds and its structure is reported here.

### Experimental

A CH_2_Cl2 solution of 5-phenyl-4,5-dihydro-1*H*-pyrazole (1.46 g, 0.01 mol)with 4-methylbenzene-1-sulfonyl chloride (2.15 g, 0.011 mol) was stired at room temperature for 4 h, then saturated aqueous sodium hydrogen carbonate (50 ml) was added into the solution. The mixture was extract with CH_2_Cl_2_. Then the solvent was removed and to give a white powder. Single crystals were obtained from the powder in methanol after 5 days.

### Refinement

H atoms were positioned geometrically (C-H = 0.93-0.98 Å) and refined using a riding model, with U_eq_(H) = 1.5U_eq_(C) for methyl group and U_eq_(H) = 1.2U_eq_(C) for others.

### Crystal data

**Table:**

C_16_H_16_N_2_O_2_S	*F*(000) = 632
*M**_r_* = 300.37	*D*_x_ = 1.318 Mg m^−^^3^
Orthorhombic, *P**c**a*2_1_	Mo *K*α radiation, λ = 0.71073 Å
Hall symbol: P 2c -2ac	Cell parameters from 2341 reflections
*a* = 19.2938 (7) Å	θ = 3.1–29.4°
*b* = 6.0438 (2) Å	µ = 0.22 mm^−^^1^
*c* = 12.9812 (5) Å	*T* = 293 K
*V* = 1513.71 (10) Å^3^	Block, colorless
*Z* = 4	0.32 × 0.28 × 0.25 mm

### Data collection

**Table:**

Oxford Diffraction Xcalibur Atlas Gemini ultra diffractometer	2168 independent reflections
Radiation source: fine-focus sealed tube	1870 reflections with *I* > 2σ(*I*)
graphite	*R*_int_ = 0.028
Detector resolution: 10.3592 pixels mm^-1^	θ_max_ = 25.4°, θ_min_ = 3.1°
ω scans	*h* = −17→23
Absorption correction: multi-scan (*CrysAlis PRO*; Oxford Diffraction, 2010)	*k* = −6→7
*T*_min_ = 0.933, *T*_max_ = 0.947	*l* = −15→10
5286 measured reflections	

### Refinement

**Table:**

Refinement on *F*^2^	Secondary atom site location: difference Fourier map
Least-squares matrix: full	Hydrogen site location: inferred from neighbouring sites
*R*[*F*^2^ > 2σ(*F*^2^)] = 0.034	H-atom parameters constrained
*wR*(*F*^2^) = 0.081	*w* = 1/[σ^2^(*F*_o_^2^) + (0.0441*P*)^2^ + 0.0462*P*] where *P* = (*F*_o_^2^ + 2*F*_c_^2^)/3
*S* = 1.05	(Δ/σ)_max_ < 0.001
2168 reflections	Δρ_max_ = 0.15 e Å^−^^3^
191 parameters	Δρ_min_ = −0.26 e Å^−^^3^
1 restraint	Absolute structure: Flack (1983), 711 Friedel pairs
Primary atom site location: structure-invariant direct methods	Flack parameter: 0.00 (8)

### Special details

**Table:**

Geometry. All e.s.d.'s (except the e.s.d. in the dihedral angle between two l.s. planes) are estimated using the full covariance matrix. The cell e.s.d.'s are taken into account individually in the estimation of e.s.d.'s in distances, angles and torsion angles; correlations between e.s.d.'s in cell parameters are only used when they are defined by crystal symmetry. An approximate (isotropic) treatment of cell e.s.d.'s is used for estimating e.s.d.'s involving l.s. planes.
Refinement. Refinement of *F*^2^ against ALL reflections. The weighted *R*-factor *wR* and goodness of fit *S* are based on *F*^2^, conventional *R*-factors *R* are based on *F*, with *F* set to zero for negative *F*^2^. The threshold expression of *F*^2^ > σ(*F*^2^) is used only for calculating *R*-factors(gt) *etc*. and is not relevant to the choice of reflections for refinement. *R*-factors based on *F*^2^ are statistically about twice as large as those based on *F*, and *R*- factors based on ALL data will be even larger.

### Fractional atomic coordinates and isotropic or equivalent isotropic displacement parameters (Å^2^)

**Table:**

	*x*	*y*	*z*	*U*_iso_*/*U*_eq_	
S1	0.36119 (3)	0.55535 (9)	0.53950 (5)	0.0517 (2)	
O1	0.37103 (10)	0.7881 (3)	0.5338 (2)	0.0737 (6)	
O2	0.34854 (11)	0.4301 (3)	0.44842 (16)	0.0680 (6)	
N1	0.29819 (12)	0.6172 (4)	0.7097 (2)	0.0680 (7)	
N2	0.29200 (10)	0.5148 (3)	0.61190 (18)	0.0511 (6)	
C1	0.43222 (13)	0.4394 (4)	0.6042 (2)	0.0452 (6)	
C2	0.45625 (14)	0.2327 (4)	0.5752 (2)	0.0555 (7)	
H2	0.4349	0.1557	0.5218	0.067*	
C3	0.51202 (15)	0.1425 (4)	0.6261 (3)	0.0581 (7)	
H3	0.5285	0.0046	0.6058	0.070*	
C4	0.54427 (13)	0.2507 (4)	0.7066 (2)	0.0547 (7)	
C5	0.60491 (17)	0.1474 (5)	0.7611 (3)	0.0828 (10)	
H5C	0.6114	0.2183	0.8266	0.124*	
H5B	0.6459	0.1653	0.7201	0.124*	
H5A	0.5961	−0.0073	0.7715	0.124*	
C6	0.51950 (14)	0.4589 (4)	0.7345 (2)	0.0530 (7)	
H6	0.5409	0.5358	0.7878	0.064*	
C7	0.46389 (13)	0.5524 (4)	0.6845 (2)	0.0500 (7)	
H7	0.4476	0.6908	0.7044	0.060*	
C8	0.26877 (16)	0.4944 (7)	0.7745 (3)	0.0833 (11)	
H8	0.2666	0.5317	0.8440	0.100*	
C9	0.23803 (18)	0.2892 (6)	0.7349 (3)	0.0839 (11)	
H9A	0.1878	0.2940	0.7383	0.101*	
H9B	0.2545	0.1614	0.7729	0.101*	
C10	0.26345 (13)	0.2845 (4)	0.6227 (2)	0.0523 (7)	
H10	0.3006	0.1753	0.6148	0.063*	
C11	0.20658 (11)	0.2411 (3)	0.5454 (2)	0.0428 (5)	
C12	0.15381 (12)	0.3928 (4)	0.5298 (3)	0.0516 (6)	
H12	0.1543	0.5262	0.5654	0.062*	
C13	0.10086 (14)	0.3487 (5)	0.4626 (2)	0.0619 (8)	
H13	0.0661	0.4530	0.4525	0.074*	
C14	0.09883 (16)	0.1535 (5)	0.4104 (3)	0.0705 (9)	
H14	0.0627	0.1240	0.3651	0.085*	
C15	0.15027 (17)	0.0006 (5)	0.4250 (3)	0.0694 (9)	
H15	0.1490	−0.1331	0.3895	0.083*	
C16	0.20389 (14)	0.0445 (4)	0.4922 (2)	0.0576 (7)	
H16	0.2387	−0.0601	0.5017	0.069*	

### Atomic displacement parameters (Å^2^)

**Table:**

	*U*^11^	*U*^22^	*U*^33^	*U*^12^	*U*^13^	*U*^23^
S1	0.0487 (4)	0.0551 (3)	0.0513 (4)	−0.0086 (3)	−0.0036 (4)	0.0102 (4)
O1	0.0702 (12)	0.0549 (9)	0.0961 (16)	−0.0138 (8)	−0.0086 (14)	0.0230 (14)
O2	0.0698 (14)	0.0891 (13)	0.0451 (12)	−0.0115 (10)	−0.0081 (10)	0.0042 (11)
N1	0.0476 (14)	0.0842 (16)	0.072 (2)	−0.0022 (12)	0.0035 (14)	−0.0205 (16)
N2	0.0439 (12)	0.0530 (10)	0.0564 (15)	−0.0063 (9)	−0.0051 (11)	0.0041 (11)
C1	0.0434 (14)	0.0462 (12)	0.0460 (16)	−0.0091 (10)	0.0061 (12)	−0.0001 (12)
C2	0.0560 (17)	0.0538 (13)	0.0567 (18)	−0.0112 (12)	0.0010 (14)	−0.0077 (14)
C3	0.0597 (17)	0.0482 (13)	0.0663 (19)	0.0048 (12)	0.0084 (16)	−0.0035 (15)
C4	0.0438 (15)	0.0616 (15)	0.0585 (18)	0.0016 (13)	0.0070 (14)	−0.0004 (16)
C5	0.068 (2)	0.091 (2)	0.089 (3)	0.0186 (18)	−0.0106 (19)	−0.003 (2)
C6	0.0498 (16)	0.0601 (15)	0.0491 (16)	−0.0071 (12)	−0.0003 (14)	−0.0072 (15)
C7	0.0482 (15)	0.0467 (12)	0.0551 (18)	−0.0020 (11)	0.0042 (13)	−0.0080 (13)
C8	0.051 (2)	0.143 (3)	0.056 (2)	−0.020 (2)	−0.0031 (17)	−0.010 (2)
C9	0.076 (2)	0.123 (3)	0.0524 (19)	−0.037 (2)	−0.0169 (18)	0.029 (2)
C10	0.0441 (14)	0.0538 (13)	0.0589 (18)	−0.0075 (11)	−0.0118 (15)	0.0135 (14)
C11	0.0375 (12)	0.0484 (11)	0.0424 (14)	−0.0041 (9)	0.0018 (14)	0.0101 (13)
C12	0.0450 (14)	0.0557 (12)	0.0540 (17)	0.0044 (10)	−0.0020 (16)	−0.0027 (15)
C13	0.0484 (16)	0.0702 (16)	0.067 (2)	0.0055 (14)	−0.0098 (16)	0.0054 (16)
C14	0.063 (2)	0.085 (2)	0.064 (2)	−0.0111 (16)	−0.0217 (16)	0.0068 (18)
C15	0.083 (2)	0.0592 (15)	0.066 (2)	−0.0050 (15)	−0.0100 (19)	−0.0082 (17)
C16	0.0557 (17)	0.0519 (14)	0.0653 (19)	0.0081 (12)	−0.0054 (15)	0.0044 (14)

### Geometric parameters (Å, °)

**Table:**

S1—O1	1.4213 (16)	C7—H7	0.9300
S1—O2	1.425 (2)	C8—C9	1.468 (5)
S1—N2	1.651 (2)	C8—H8	0.9300
S1—C1	1.754 (3)	C9—C10	1.537 (5)
N1—C8	1.257 (4)	C9—H9A	0.9700
N1—N2	1.417 (3)	C9—H9B	0.9700
N2—C10	1.504 (3)	C10—C11	1.510 (4)
C1—C2	1.385 (3)	C10—H10	0.9800
C1—C7	1.387 (4)	C11—C16	1.375 (4)
C2—C3	1.376 (4)	C11—C12	1.385 (3)
C2—H2	0.9300	C12—C13	1.370 (4)
C3—C4	1.380 (4)	C12—H12	0.9300
C3—H3	0.9300	C13—C14	1.361 (4)
C4—C6	1.394 (3)	C13—H13	0.9300
C4—C5	1.503 (4)	C14—C15	1.369 (4)
C5—H5C	0.9600	C14—H14	0.9300
C5—H5B	0.9600	C15—C16	1.379 (4)
C5—H5A	0.9600	C15—H15	0.9300
C6—C7	1.376 (4)	C16—H16	0.9300
C6—H6	0.9300		
			
O1—S1—O2	120.35 (16)	N1—C8—C9	116.5 (3)
O1—S1—N2	106.53 (13)	N1—C8—H8	121.7
O2—S1—N2	104.77 (12)	C9—C8—H8	121.7
O1—S1—C1	108.43 (12)	C8—C9—C10	102.6 (3)
O2—S1—C1	108.62 (12)	C8—C9—H9A	111.2
N2—S1—C1	107.45 (11)	C10—C9—H9A	111.2
C8—N1—N2	107.7 (3)	C8—C9—H9B	111.2
N1—N2—C10	110.6 (2)	C10—C9—H9B	111.2
N1—N2—S1	112.15 (17)	H9A—C9—H9B	109.2
C10—N2—S1	119.12 (17)	N2—C10—C11	111.4 (2)
C2—C1—C7	120.1 (2)	N2—C10—C9	100.8 (2)
C2—C1—S1	119.4 (2)	C11—C10—C9	113.7 (2)
C7—C1—S1	120.48 (18)	N2—C10—H10	110.2
C3—C2—C1	119.2 (3)	C11—C10—H10	110.2
C3—C2—H2	120.4	C9—C10—H10	110.2
C1—C2—H2	120.4	C16—C11—C12	118.1 (2)
C2—C3—C4	121.9 (2)	C16—C11—C10	120.7 (2)
C2—C3—H3	119.0	C12—C11—C10	121.1 (2)
C4—C3—H3	119.0	C13—C12—C11	120.9 (2)
C3—C4—C6	118.0 (3)	C13—C12—H12	119.6
C3—C4—C5	120.7 (2)	C11—C12—H12	119.6
C6—C4—C5	121.3 (3)	C14—C13—C12	120.4 (3)
C4—C5—H5C	109.5	C14—C13—H13	119.8
C4—C5—H5B	109.5	C12—C13—H13	119.8
H5C—C5—H5B	109.5	C13—C14—C15	119.7 (3)
C4—C5—H5A	109.5	C13—C14—H14	120.1
H5C—C5—H5A	109.5	C15—C14—H14	120.1
H5B—C5—H5A	109.5	C14—C15—C16	120.1 (3)
C7—C6—C4	121.0 (3)	C14—C15—H15	120.0
C7—C6—H6	119.5	C16—C15—H15	120.0
C4—C6—H6	119.5	C11—C16—C15	120.8 (3)
C6—C7—C1	119.7 (2)	C11—C16—H16	119.6
C6—C7—H7	120.1	C15—C16—H16	119.6
C1—C7—H7	120.1		

## References

[bb1] Dolomanov, O. V., Bourhis, L. J., Gildea, R. J., Howard, J. A. K. & Puschmann, H. (2009). *J. Appl. Cryst.* **42**, 339–341.

[bb2] Flack, H. D. (1983). *Acta Cryst.* A**39**, 876–881.

[bb3] Goodell, J. R., Puig-Basagoiti, F., Forshey, B. M., Shi, P. Y. & Ferguson, D. M. (2006). *J. Med. Chem.* **49**, 2127–2137. 10.1021/jm051229y 16539402

[bb4] Oxford Diffraction (2010). *CrysAlis PRO* Oxford Diffraction Ltd, Yarnton, England.

[bb5] Park, H. J., Lee, K., Park, S. J., Ahn, B., Lee, J. C., Cho, H. Y. & Lee, K. I. (2005). *Bioorg. Med. Chem. Lett.* **15**, 3307–3312. 10.1016/j.bmcl.2005.03.082 15922597

[bb6] Shaharyar, M., Siddiqui, A. A. & Ali, M. A. (2006). *Bioorg. Med. Chem. Lett.* **16**, C4571–C4574. 10.1016/j.bmcl.2006.06.021 16784842

[bb7] Sheldrick, G. M. (2008). *Acta Cryst.* A**64**, 112–122. 10.1107/S0108767307043930 18156677

[bb8] Suresh, K., Sandhya, B., Sushma, D., Rajiv, K. & Himanshu, G. (2009). *Recent Patents Anti-Infect. Drug Disc.* **4**, 154–163.

